# Strain Localization of Orthotropic Elasto–Plastic Cohesive–Frictional Materials: Analytical Results and Numerical Verification

**DOI:** 10.3390/ma14082040

**Published:** 2021-04-18

**Authors:** Sungchul Kim, Miguel Cervera, Jian-Ying Wu, Michele Chiumenti

**Affiliations:** 1 Campus Norte, CIMNE, Technical University of Catalonia, Edificio C1, Jordi Girona 1-3, 08034 Barcelona, Spain; Sungchul.kim@upc.edu (S.K.); michele.chiumenti@upc.edu (M.C.); 2 State Key Laboratory of Subtropical Building Science, South China University of Technology, Guangzhou 510641, China; Jywu@scut.edu.cn

**Keywords:** localized failure, strain localization, orthotropic plasticity, cohesive–frictional materials, plasticity

## Abstract

Strain localization analysis for orthotropic-associated plasticity in cohesive–frictional materials is addressed in this work. Specifically, the localization condition is derived from Maxwell’s kinematics, the plastic flow rule and the boundedness of stress rates. The analysis is applicable to strong and regularized discontinuity settings. Expanding on previous works, the quadratic orthotropic Hoffman and Tsai–Wu models are investigated and compared to pressure insensitive and sensitive models such as von Mises, Hill and Drucker–Prager. Analytical localization angles are obtained in uniaxial tension and compression under plane stress and plane strain conditions. These are only dependent on the plastic potential adopted; ensuing, a geometrical interpretation in the stress space is offered. The analytical results are then validated by independent numerical simulations. The B-bar finite element is used to deal with the limiting incompressibility in the purely isochoric plastic flow. For a strip under vertical stretching in plane stress and plane strain as well as Prandtl’s problem of indentation by a flat rigid die in plane strain, numerical results are presented for both isotropic and orthotropic plasticity models with or without tilting angle between the material axes and the applied loading. The influence of frictional behavior is studied. In all the investigated cases, the numerical results provide compelling support to the analytical prognosis.

## 1. Introduction

Orthotropic Materials such as wood and masonry have been traditionally used in construction and are very much used today. Other frequently used materials, such as rolled metals, are orthotropic because of their manufacturing process. This is also very much the case of metallic and polymeric materials and components produced layer-by-layer using modern additive manufacturing (AM) techniques, now increasingly used. In the field of geological engineering, the analysis of orthotropic materials is of interest in ground excavation, tunnel construction and landslides prevention.

Hill [1,2,3], a pioneer in the mathematical research of plasticity, proposed several constitutive orthotropic plasticity models for sheet metals and investigated strain localization and failure of orthotropic plastic materials. Based on Hill’s works, many isotropic and orthotropic plastic criteria have been later proposed, such as the Drucker–Prager model [4,5,6,7], Hoffman model [8], Tsai–Wu model [9], and many more [10,11,12]. Purely cohesive models that are insensitive to pressure and yield an isochoric plastic flow, such as the von Mises and the Hill models, are appropriate for metallic materials. Associated elasto-plastic cohesive–frictional models such as the Drucker–Prager, Hoffman and Tsai–Wu models are suitable for simulating polymeric materials such as PVC H100, H250 and carbon fiber composites [13,14,15,16] with isotropic and orthotropic behavior, as these materials show distinct strengths under tensile and compressive loading. Additive manufacturing techniques based on filament deposition or powder bed fusion introduce different levels of orthotropy in the mechanical stiffness and strength of the fabricated components. Geomaterials like soils, concrete, masonry and rocks are also modeled with pressure-sensitive plasticity models; non-associated plasticity is also used for these materials in order to better approximate the real dilatant behavior.

In plastic materials subjected to increasing loading beyond yielding, plastic strains tend to concentrate in narrow zones called shear bands. This phenomenon, consisting of irreversible deformation concentrating in a definite thin zone, is known as strain localization [17]. Strain localization results in strain (weak) discontinuities across the surfaces limiting the shear band. If the size of the band is very small compared to the dimensions of the plastic medium, the band appears as (strong) discontinuity surface across which the displacement field is discontinuous.

Structural assessment requires the accurate prediction of failure mechanisms and peak carrying loads. Thus, failure mechanics has evolved in the last decades as a very active field, and much analytical, experimental and computational research effort has been invested in plasticity, damage, and fracture mechanics. Lately, computational failure mechanics has often addressed the problem of the phenomenon of plastic strain localization and the analytical and numerical challenges associated to it.

Early works of Prandtl [18], Hencky [19,20], and Mandel [21] determined the directions of the slip lines, and the associated failure mechanisms and loads. Hill revisited and interpreted the slip lines as the characteristic lines of the hyperbolic plastic governing equations assuming rigid-plastic and incompressible behavior preceding shear-driven plastic yielding.

Hill [22,23], Thomas [24] and Rice [25] investigated strain localization as a bifurcation problem and extended the scope from rigid-plastic to elasto-plastic solids. Rice [26] extended the plane strain slip line theory to anisotropic rigid-plastic material. Rudnicki and Rice [27] investigated the localization of deformation of pressure-sensitive dilatant materials such as brittle rocks. Pietruszczak and Mróz [28] studied strain-softening in isotropic Coulomb elasto-plastic materials. Nielsen and Schreyer [29] studied the loss of strong ellipticity in associated and non-associated elasto-plasticity. Strain localization in frictional solids was researched by Leroy and Ortiz [30]. Forest [31] used continuum models for strain localization in metallic foams. Borja [32,33] extended the modeling in elasto-plastic models and soft rocks. Willam and coworkers [34,35] studied the localization properties of standard and generalized Drucker–Prager models. Vrech [36] addressed localization analysis of gradient-dependent parabolic Drucker–Prager models. Zhang [37] studied damage and strain localization in geomaterials, and Tasan [38] studied strain localization and damage in dual phase steels.

The classical bifurcation analysis has been applied to both weak and strong discontinuities. Simo [39] and Oliver [40,41] studied the orientation of strong discontinuities in inelastic solids, and Oliver [42,43] suggested continuum plasticity models for the modeling of such strong discontinuities. They soon found that conditions for discontinuous bifurcation do not necessarily guarantee the occurrence of strong discontinuities, unless the strong discontinuity is properly regularized and stress boundedness is invoked [40,41,42,43].

The authors [44,45] used Maxwell’s compatibility condition and stress boundedness to predict analytically the orientation of shear discontinuities for isotropic von Mises and orthotropic Hill elasto-plastic models. The analytical results were verified numerically. This strain localization analysis was successfully applied to other and elastic-damage models [46,47,48].

It turns out that the stress boundedness condition is a more constrictive necessary condition than the classical discontinuous bifurcation condition, as strain localization generally occurs after strain bifurcation has occurred. Contrary to the strain bifurcation conditions, this strain localization condition depends entirely on the inelastic flow; remarkably, it does not depend on the elastic properties or on the yield surface. The localization angles can be analytically predicted from the inelastic flow tensor alone.

This paper addresses the analytical determination of the orientation of slip lines in orthotropic elasto-plastic cohesive–frictional materials by extending the strain localization analysis developed in previous works. The objectives are fourfold: (i) to extend the strain localization analysis to orthotropic elasto-plastic cohesive–frictional materials; (ii) to derive analytically localization angles in plane stress and plane strain conditions for these models; (iii) to verify these analytical results via independent numerical simulations; and (iv) to investigate the influence of plastic material properties on strain localization in orthotropic cohesive–frictional materials.

The paper is structured as follows. Section 2 briefly presents the analytical framework: constitutive relations, kinematics for strong and weak discontinuities, and strain localization conditions. Section 3 introduces orthotropic plasticity and develops the analytical results for the localization angles in plane stress and plane strain conditions with some examples. In Section 4, numerical verification of the analytical results using B-bar finite elements is offered. Section 5 closes the paper with some conclusions.

## 2. Strain Localization in Elasto-Plastic Solids

In this section, the mechanics of strain localization in elasto-plastic media is addressed. Using Maxwell’s kinematics and assuming boundedness of the stress rates, the necessary condition for strain localization in elasto-plastic materials is obtained. The results hold both for strong (displacement) discontinuities and for regularized strain localization bands limited by weak (strain) discontinuities.

Let $\Omega \subset R_{\;}^{n_{dim}}$ ($n_{\dim}=1,\;2,\;3$) be an elasto-plastic solid domain, with the reference position vector $x\subset R_{\;}^{n_{dim}}$. The outer boundary is denoted by $\Gamma \subset R_{\;}^{n_{dim}}{}^{-1}$, with the outward unit normal vector $n^{*}$. Deformations of the solid are characterized by the displacement field u(x) and the infinitesimal strain field $\epsilon \left(x\right)=\nabla^{\mathrm{sym}}u\left(x\right)$, where $\nabla^{\mathrm{sym}}\left(\;\cdot \;\right)$ is the symmetric gradient operator.

### 2.1. Elasto-Plasticity Model

In the following, tensorial notation is used. The inner products with single and double contractions are denoted by ‘·’ and ‘:’, respectively, while the dyadic operator is signified by ‘⨂’.

For the elasto-plastic model, the constitutive equation is expressed in total form as

(1) $$\epsilon =\epsilon^{e}+\epsilon^{p},\;\;\;\sigma =E^{0}:\epsilon^{e}=E^{0}:\left(\epsilon -\epsilon^{p}\right)$$

where the second-order strain tensor **ϵ** is decomposed into its elastic and plastic parts, $\epsilon^{e}$ and $\epsilon^{p}$. The second-order stress tensor σ is proportional to the elastic strain tensor$\;\epsilon^{e}$, through the fourth-order elasticity tensor $E^{0}$. All the tensors involved are symmetric. The elastic properties may be orthotropic.

The admissible stress domain is determined by the yield criterion Φ(σ,ζ)=ϕ(σ)−q(ζ)≤ 0, defined in terms of the equivalent stress ϕ(σ) and a stress-like internal variable q(ζ), which determine the shape and size of the domain, respectively. Yield criteria for orthotropic elasto-plasticity are discussed in Section 3.

The plastic strain is defined in rate form, its direction is derived from a plastic potential. In associated plasticity, the plastic potential is equal to the yield surface, so that

(2) $${\dot{\epsilon }}^{p}=\;\dot{\lambda }\;\frac{\partial \phi }{\partial \sigma }=\;\dot{\lambda }\;\;\Lambda$$

where $\;\dot{\lambda }\;\geq 0$ denotes the plastic multiplier; $\dot{⬚}$ is the time derivative and the plastic flow tensor Λ=∂ϕ /∂σ is normal to the yield surface Φ=0. Similarly, the evolution of the size of the yield surface is determined by

(3) $$\;\dot{\zeta }\;=\;\dot{\lambda }\;\frac{\partial \phi }{\partial q}=-\;\dot{\lambda }\;$$

The constitutive equation in rate form follows from Equation (1),

(4) $$\dot{\sigma }=E^{0}:{\dot{\epsilon }}^{e}=E^{0}:\left(\dot{\epsilon }-{\dot{\epsilon }}^{p}\right)=E^{\mathrm{ep}}:\dot{\epsilon }$$

where the fourth-order elasto-plasticity tangent tensor $E^{\mathrm{ep}}$ is obtained from the Kuhn–Tucker and consistency conditions as

(5) $$E^{\mathrm{ep}}=\frac{d\sigma }{d\epsilon }=E^{0}-\frac{E^{0}:\Lambda ⨂E^{0}:\Lambda }{H+\Lambda :E^{0}:\Lambda }$$

where H=∂q /∂ζ is the hardening or softening modulus. For perfect plasticity, $q=q_{0}$, and H=0. Note that in associated plasticity, the elasto-plastic tangent tensor is symmetric.

### 2.2. Kinematics of Strong and Regularized Discontinuities

In the early stages of the loading and deformation process of an elasto-plastic solid, standard kinematics applies and both the displacement rate and strain rate fields are continuous. However, in softening and associated perfect plasticity, and even in hardening non-associated plasticity, slip lines (in 2D) or slip surfaces (in 3D) may form. Across these, the deformation can grow unbounded, displacement and/or strain discontinuities may appear and Maxwell’s compatibility condition needs to be considered.

**Figure 1:**
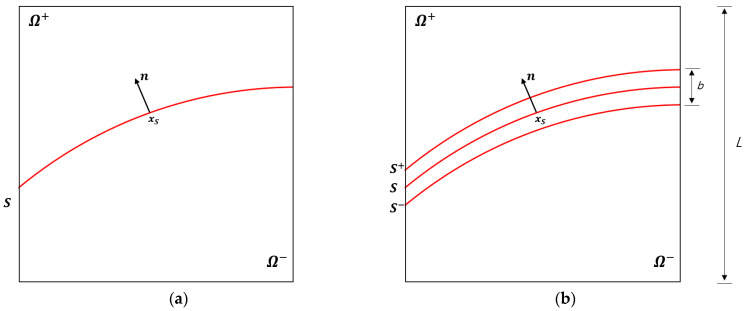
(**a**) Strong and (**b**) regularized discontinuities in an elasto-plastic solid.

Figure 1a shows the elasto-plastic solid domain Ω split by a displacement discontinuity S (the slip line or slip surface) into two parts $\Omega^{+}$ and $\Omega^{-}$. The orientation of the discontinuity is denoted with the unit normal vector n with direction from $\Omega^{-}$ to $\Omega^{+}$. Let *L* be a characteristic size of the domain.

**Figure 2:**
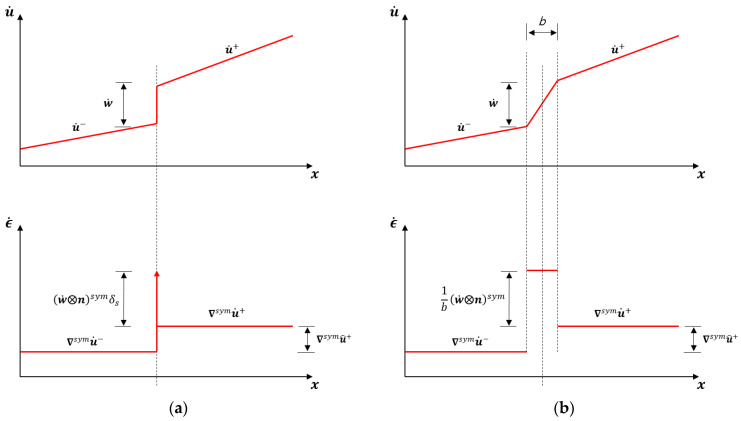
Kinematics of (**a**) strong and (**b**) regularized discontinuities.

Figure 2a shows the corresponding kinematics: the velocity and strain rate fields are not regular. There is a discontinuity of the displacement rate at S of value $\dot{w}$; correspondingly, the strain rate at S is

(6) $${\dot{\epsilon }}_{S}={\left(\dot{w}\;⨂\;n\right)}^{\mathrm{sym}}\delta_{S}$$

where $\delta_{S}$ denotes the Dirac delta function. Note that this strain rate is unbounded and has a very definite structure determined by the direction of the discontinuity surface, as it allows for unbounded strain rate components at S due to the discontinuity of the displacement in the normal direction n, but not in those directions tangential to S.

For the analysis of strain localization in the continuum setting and also for its numerical verification using FEM, it is convenient to consider a *regularized* discontinuity, as shown in Figure 1b. Here, subdomains $\Omega^{+}$ and $\Omega^{-}$ are separated by a regularized discontinuity band B of finite width *b*, as the distance between surfaces $S^{+}$ and $S^{-}$; these are weak (strain) discontinuities. The bandwidth *b* is small compared to the characteristic size of the domain *L*, so that b/L<<1.

Figure 2b shows the corresponding regularized kinematics. Note that the strain localizes in the regularized band B. The deformation rate vector in the strain localization band $\dot{e}\;$is defined as the (apparent) jump of displacement rate $\dot{w}$ across the regularized discontinuity band divided by the band width, $\dot{e}=\dot{w}/b$.

Let $\dot{u}$ be a characteristic displacement in domain and the jump $\dot{w}$ be of the same order. Deformations outside the localization band are of the order ${\dot{e}}_{\mathrm{ext}}=\dot{u}/L$, while inside the band they are of order ${\dot{e}}_{\mathrm{int}}=\dot{w}/L$. As b/L<<1, ${\dot{e}}_{\mathrm{ext}}/{\dot{e}}_{\mathrm{int}}<<1$ even for a finite, small bandwidth.

Denoting by ${\dot{\epsilon }}_{\mathrm{ext}}$ and ${\dot{\epsilon }}_{\mathrm{int}}$ the strain rates inside and outside of the localization band, respectively, and being $⟦\dot{\epsilon }⟧\;\mathrm{the}\;\mathrm{corresponding}\;\mathrm{strain}\;\mathrm{rate}\;\mathrm{jump},\;$Maxwell’s compatibility condition [20] is now expressed as

(7) $$⟦\dot{\epsilon }⟧={\dot{\epsilon }}_{\mathrm{int}}-{\dot{\epsilon }}_{\mathrm{ext}}={\left(\dot{e}⨂n\right)}^{\mathrm{sym}}$$

Equation (7) is the regularized counterpart of Equation (6). Note that for the band width *b* → 0, the strain rate in the regularized discontinuity band B tends to the strain rate in the strong discontinuity S.

### 2.3. Strain Localization and Stress Boundedness

Upon strain localization inside the band, and ongoing deformation, the deformation vector rate in the band, $\dot{e}=\dot{w}/b,$ the strain rate jump, ${\left(\dot{e}\;⨂\;n\right)}^{\mathrm{sym}}$, and the plastic strain rate in the band, ${\dot{\epsilon }}_{\mathrm{int}}^{p}$, will grow much larger than the total strain rate outside the band,$\;{\dot{\epsilon }}_{\mathrm{ext}}$, and the corresponding plastic strain rate,$\;{\dot{\epsilon }}_{\mathrm{ext}}^{p}$, will either vanish (on elastic unloading) or remain small (on plastic loading); this ensures boundedness of the stress rate outside the band, ${\dot{\sigma }}_{\mathrm{ext}}$. The terms that will grow upon strain localization, inversely proportional to *b*, are underlined in the following.

From the constitutive relation of the elasto-plastic solids, the stress rates inside and outside of the localization band are given by

(8) $${\dot{\sigma }}_{\mathrm{int}}=E^{0}:\left(\underline{{\dot{\epsilon }}_{\mathrm{int}}}-\underline{{\dot{\epsilon }}_{\mathrm{int}}^{p}}\right),\;{\dot{\sigma }}_{\mathrm{ext}}=E^{0}:\left({\dot{\epsilon }}_{\mathrm{ext}}-{\dot{\epsilon }}_{\mathrm{ext}}^{p}\right)$$

Note that plastic behavior is considered inside and outside the localization band. The jump of stress rate $⟦\dot{\sigma }⟧$ is expressed as

(9) $$⟦\dot{\sigma }⟧={\dot{\sigma }}_{\mathrm{int}}-{\dot{\sigma }}_{\mathrm{ext}}=E^{0}:\left(\underline{⟦\dot{\epsilon }⟧}-\underline{⟦{\dot{\epsilon }}^{p}⟧}\;\right)=E^{0}:\left[{\underline{\left(\dot{e}\;⨂\;n\right)}}^{\mathrm{sym}}-\underline{⟦{\dot{\epsilon }}^{p}⟧}\right]$$

where the compatibility Condition (7) has been used and the jump of plastic strain rate is

(10) $$\underline{⟦{\dot{\epsilon }}^{p}⟧}=\underline{{\dot{\epsilon }}_{\mathrm{int}}^{p}}-{\dot{\epsilon }}_{\mathrm{ext}}^{p}=\underline{{\;\dot{\lambda }\;}_{\mathrm{int}}}\;\Lambda -{\;\dot{\lambda }\;}_{\mathrm{ext}}\;\Lambda =\underline{\;⟦\dot{\lambda }⟧\;}\;\Lambda$$

Equations (8)–(10) are derived from the constitutive behavior and the compatibility conditions across the weak discontinuities $S^{+}$ and $S^{-}$; as strain localization has not been invoked, all the terms involved are bounded.

Inside the localization band, elasto-plastic behavior and satisfaction of the yield criterion ensure that the stress rate needs to remain bounded even if the strain rate is not. Consequently, the jump of the stress rate in Equation (9) may not be null, but it is bounded; therefore, stress rate boundedness requires that

(11) $$⟦\dot{\epsilon }⟧={\left(\dot{e}\;⨂\;n\right)}^{\mathrm{sym}}={\dot{\epsilon }}_{\mathrm{int}}^{p}={\;\dot{\lambda }\;}_{\mathrm{int}}\;\Lambda$$

The entire jump of the strain rate is due to the plastic strain rate inside the band. This a necessary condition for strain localization to occur. Some Remarks are in order.

**Remark** **1.** *This condition holds for small finite bandwidths b, as in regularized discontinuities and standard FEM simulations. The condition for strong discontinuities follows for the limit case of vanishing bandwidth b → 0*.

**Remark** **2.** *This condition does not necessarily occur upon plastic yielding or strain bifurcation. Therefore, a transition stage may be necessary in most situations during which plastic behavior happens without strain localization. Only when the localization condition is fulfilled, might true strain localization happen*.

**Remark** **3.** *Only kinematic conditions depending on the plastic flow rule are implied; therefore, the condition may be extended to non-associated plasticity*.

**Remark** **4.** *For the same reason, the condition is independent of the elastic properties. Application to rigid-plastic materials can be implied from this independence. This is not the case for classical conditions related to strain bifurcation*.

**Remark** **5.** *Stress rate continuity upon strain localization follows from Equation (9) if unloading occurs outside the band, that is, ${\dot{\lambda }}_{ext}=0$. In this case, $⟦\dot{\epsilon }⟧=⟦{\dot{\epsilon }}^{p}⟧\;and\;⟦\dot{\sigma }⟧=0.$ This is usually the case when softening plasticity is considered*.

### 2.4. Strain Localization Plastic Flow Vector and Tensor

In the following, the subscript ${\left(⬚\right)}_{\mathrm{int}}\;$will be omitted for the sake of simplicity, as all quantities refer to points inside the localization band. From Equation (10), a plastic flow localization vector, γ, can be defined so that the deformation rate vector $\dot{e}$ and the plastic flow tensor Λ are written as

(12) $$\dot{e}=\;\dot{\lambda }\;\gamma ,\;\;\;\;\;\;\;\;\;\;\;\;\;\Lambda ={\left(\gamma \;⨂\;n\right)}^{\mathrm{sym}}$$

where n is the unit vector normal to the discontinuity S. Note that Λ is a second order tensor, while $\dot{e}$, γ and n are vectors.

Let m and p be unit vectors on the plane of the discontinuity S such that (n,m,p) is a basis of orthonormal vectors. Then, the plastic flow localization vector, γ, can be equivalently defined so that

(13) $$\gamma =2n\cdot \Lambda -\Lambda_{nn}n=\gamma_{n}n+\gamma_{m}m+\gamma_{p}$$

The components of the plastic flow localization vector γ= ($\gamma_{n},\gamma_{m},\gamma_{p}$) are determined

so that

(14) $$\gamma_{n}=\gamma \cdot n=\Lambda_{nn},\;\gamma_{m}=\gamma \cdot m=2\Lambda_{nm},\;{\;\gamma }_{p}=\gamma \cdot p=2\Lambda_{np}$$

(15) $$\gamma =\Lambda_{nn}n+2\Lambda_{nm}m+2\Lambda_{np}p$$

Accordingly, the other components of the strain localization plastic flow tensor are zero:

(16) $$\Lambda_{mm}=0,\;\;\;\;\Lambda_{pp}=0,\;\;\;\Lambda_{mp}=0$$

From these equations the orientation of the slip surface may be derived.

## 3. Application to Orthotropic Cohesive–Frictional Plastic Materials

In this section, the above results for strain localization in elasto-plastic materials are purposedly applied to orthotropic cohesive–frictional plastic materials. A general form of the considered yield criteria is given that allows closed-form solutions for the orientation of the slip lines in 2D plane strain and plane stress conditions.

### 3.1. Orthotropic Cohesive–Frictional Plasticity

Orthotropic cohesive–frictional yield criteria of the form Φ(σ,ζ)=ϕ(σ)−q(ζ)≤ 0 are now considered. Let (1,2,3) be the material orthotropy axes and

(17) $$\sigma^{T}=\left[\sigma_{11},\sigma_{22},\sigma_{33},\sigma_{12},\sigma_{13},\sigma_{23}\right]$$

Voigt’s representation of the symmetric second-order stress tensor is used in those axes. Voigt’s notation will be used in the following for symmetric second-order tensors. The equivalent stress ϕ(σ) is expressed as

(18) $$\phi \left(\sigma \right)=\sqrt{\frac{3}{2}\left(\sigma^{T}\cdot P\cdot \sigma +Q^{T}\cdot \sigma \right)}$$

The generalized orthotropic matrix P and **Q** vectors read

(19) $$P=\frac{1}{F+G+H}\left[\begin{array}{cccccc} F+G & -\tilde{F} & -\tilde{G} & 0 & 0 & 0\\ -\tilde{F} & F+H & -\tilde{H} & 0 & 0 & 0\\ -\tilde{G} & -\tilde{H} & G+H & 0 & 0 & 0\\ 0 & 0 & 0 & 2L & 0 & 0\\ 0 & 0 & 0 & 0 & 2M & 0\\ 0 & 0 & 0 & 0 & 0 & 2N \end{array}\right],Q=\frac{1}{F+G+H}\left[\begin{array}{c} I\\ J\\ K\\ 0\\ 0\\ 0 \end{array}\right]$$

where the material parameters F*,* G*,* H*,* $\tilde{F}$*,* $\tilde{G}$*,* $\tilde{H}$*, L, M, N, I, J* and *K* are given in terms of the material strengths (superscripts *c* and *t* denote compression and tension, respectively):

(20) $$\begin{array}{c} F=\frac{1}{2}[\frac{1}{f_{1}^{c}f_{1}^{t}}+\frac{1}{f_{2}^{c}f_{2}^{t}}-\frac{1}{f_{3}^{c}f_{3}^{t}}],\;G=\frac{1}{2}[\frac{1}{f_{1}^{c}f_{1}^{t}}-\frac{1}{f_{2}^{c}f_{2}^{t}}+\frac{1}{f_{3}^{c}f_{3}^{t}}],\;\;\\ H=\frac{1}{2}[-\frac{1}{f_{1}^{c}f_{1}^{t}}+\frac{1}{f_{2}^{c}f_{2}^{t}}+\frac{1}{f_{3}^{c}f_{3}^{t}}] \end{array}$$

(21) $$L=\frac{1}{2}{\left(\frac{1}{f_{12}}\right)}^{2},\;M=\frac{1}{2}{\left(\frac{1}{f_{13}}\right)}^{2},\;N=\frac{1}{2}{\left(\frac{1}{f_{23}}\right)}^{2}$$

(22) $$I=\frac{1}{f_{1}^{t}}-\frac{1}{f_{1}^{c}},\;J=\frac{1}{f_{2}^{t}}-\frac{1}{f_{2}^{c}},\;K=\frac{1}{f_{3}^{t}}-\frac{1}{f_{3}^{c}}$$

Unless otherwise stated:

(23) $$\tilde{F\;}=F,\;\;\;\;\;\;\tilde{G\;}=G,\;\;\;\;\;\;\tilde{H}=H$$

The initial stress threshold is defined as

(24) $$q_{0}^{2}=\frac{3}{2}{\left[F+G+H\right]}^{-1}$$

Different well-known quadratic isotropic and orthotropic yield criteria are obtained by appropriately selecting the material parameters:

Von Mises criterion:

(25) $$f=f_{1}^{c}=f_{2}^{c}=f_{3}^{c}=f_{1}^{t}=f_{2}^{t}=f_{3}^{t},\;\;\;\;\;\;\;\frac{f}{\sqrt{3}}=f_{12}=f_{13}=f_{23}$$

Parabolic Drucker–Prager (DP) criterion:

(26) $$f^{c}=f_{1}^{c}=f_{2}^{c}=f_{3}^{c},\;f^{t}=f_{1}^{t}=f_{2}^{t}=f_{3}^{t},\;\frac{\sqrt{f^{c}f^{t}}}{\sqrt{3}}=f_{12}=f_{13}=f_{23}$$

Hill criterion:

(27) $$f_{1}=f_{1}^{c}=f_{1}^{t},f_{2}=f_{2}^{c}=f_{2}^{t},f_{3}=f_{3}^{c}=f_{3}^{t}\;\mathrm{and}\;I=J=K=0$$

Hoffman criterion:

(28) $$\tilde{F\;}=F,\tilde{G\;}=G,\tilde{H}=H$$

Tsai–Wu criterion:

(29) $$\tilde{F}=\frac{1}{2}\frac{1}{\sqrt{f_{1}^{c}f_{1}^{t}f_{2}^{c}f_{2}^{t}}}\;,\;\;\tilde{G}=\frac{1}{2}\frac{1}{\sqrt{f_{1}^{c}f_{1}^{t}f_{3}^{c}f_{3}^{t}}},\;\;\tilde{H}=\frac{1}{2}\frac{1}{\sqrt{f_{2}^{c}f_{2}^{t}f_{3}^{c}f_{3}^{t}}}$$

**Remark** **6.** *The effective stress in Equation (18) defines a quadratic yield surface, with a quadratic dependence of the friction-angle on pressure. Alternatively, an effective stress defined as*

(30) $$\phi \left(\sigma \right)=\sqrt{\frac{3}{2}\sigma^{T}\cdot P\cdot \sigma }+{\hat{Q}}^{T}\cdot \sigma$$

with ${\hat{Q}}_{i}=\sqrt{Q_{i}}\;$allowing for a yield surface with straight meridians; the isotropic criterion would the more conventional DP cone.

**Figure 3:**
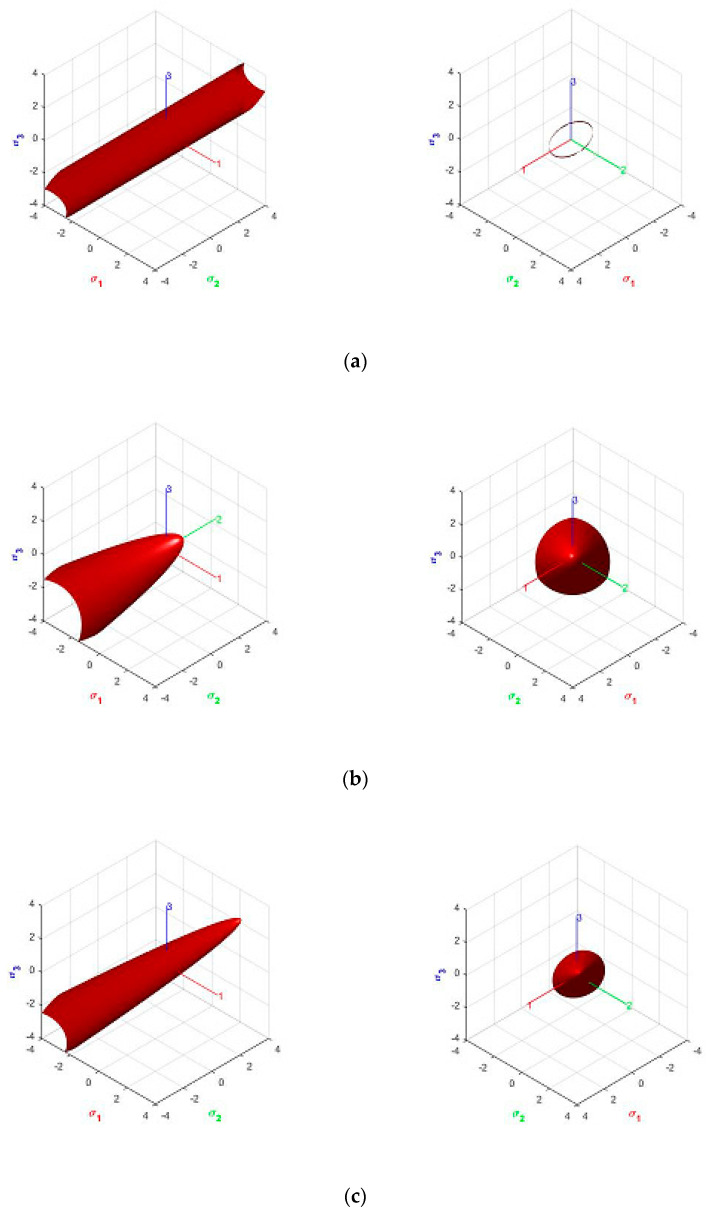

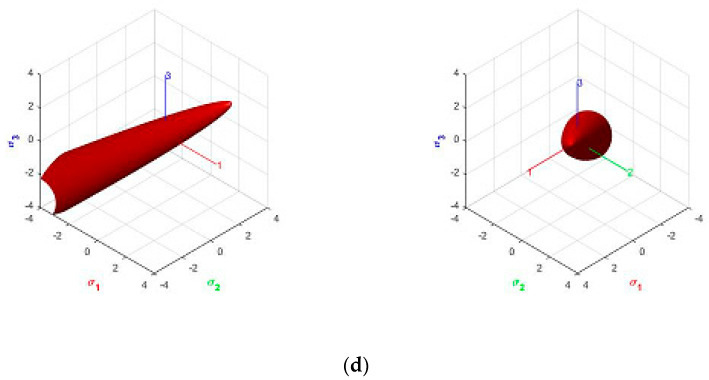
Yield criteria in Haigh–Westergaard (HW) stress space, lateral view from the hydrostatic axis: (**a**) Hill; (**b**) Parabolic Drucker–Prager; (**c**) Hoffman; (**d**) Tsai–Wu.

Orthotropic criteria cannot be represented graphically in the Haigh–Westergaard (HW) stress space because they depend on the six stress components. A partial graphical representation can be obtained by considering them projected into the HW space when the principal stresses act on the material axis, that is, no shear stress appears on the material system. Such representation, generally as an elliptic paraboloid, is offered in Figure 3. All strengths are scaled to 1. Figure 3a shows an orthotropic Hill cylinder, with $f_{1}/\;f_{2}=f_{1}/\;f_{3}=1.5$, tensile and compresive strenth are equal. Figure 3b show the isotropic parabolic Drucker–Prager for compressive to tensile strength ratio $\kappa =f^{c}/\;f^{t}=1.5$. Figure 3d,c show the orthotropic Hoffman and Tsai–Wu criteria, respectively, for ratios $\kappa =f_{1}^{c}\;/f_{1}^{t}=1.5$ and $f_{2}^{c}\;/f_{2}^{t}=f_{3}^{c}\;/f_{3}^{t}=1;\;$all tensile strengths are taken equal to 1.

### 3.2. Orthotropic Plastic Flow

From the effective stress in Equation (18), the components of the plastic flow tensor plastic flow are obtained:

(31) $$\Lambda =\frac{\partial \phi }{\partial \sigma }=\frac{3}{4}\frac{1}{\phi \left(\sigma \right)}\;\left(2\;P\cdot \sigma +Q^{T}\right)$$

So that

(32) $$\Lambda_{11}=\frac{\partial \phi }{\partial \sigma_{11}}=\frac{q_{0}^{2}}{2\phi }\left[2\left(G+F\right)\sigma_{11}-2\tilde{F}\sigma_{22}-2\tilde{G}\sigma_{33}+I\right]$$

(33) $$\Lambda_{22}=\frac{\partial \phi }{\partial \sigma_{22}}=\frac{q_{0}^{2}}{2\phi }\left[2\left(F+H\right)\sigma_{22}-2\tilde{F}\sigma_{11}-2\tilde{H}\sigma_{33}+J\right]$$

(34) $$\Lambda_{33}=\frac{\partial \phi }{\partial \sigma_{33}}=\frac{q_{0}^{2}}{2\phi }\left[2\left(G+H\right)\sigma_{33}-2\tilde{G}\sigma_{11}-2\tilde{H}\sigma_{22}+K\right]$$

(35) $$\Lambda_{12}=\Lambda_{21}=\frac{1}{2}\frac{\partial \phi }{\partial \sigma_{12}}=\frac{q_{0}^{2}}{2\phi }L\sigma_{12}$$

(36) $$\Lambda_{13}=\Lambda_{31}=\frac{1}{2}\frac{\partial \phi }{\partial \sigma_{13}}=\frac{q_{0}^{2}}{2\phi }M\sigma_{13}$$

(37) $$\Lambda_{23}=\Lambda_{32}=\frac{1}{2}\frac{\partial \phi }{\partial \sigma_{23}}=\frac{q_{0}^{2}}{2\phi }N\sigma_{23}$$

The identity $\mathrm{tr}\Lambda =\Lambda_{11}+\Lambda_{22}+\Lambda_{33}=\frac{q_{0}^{2}}{2\phi }\left(I+J+K\right)$ holds.

### 3.3. Strain Localization Angle

**Figure 4:**
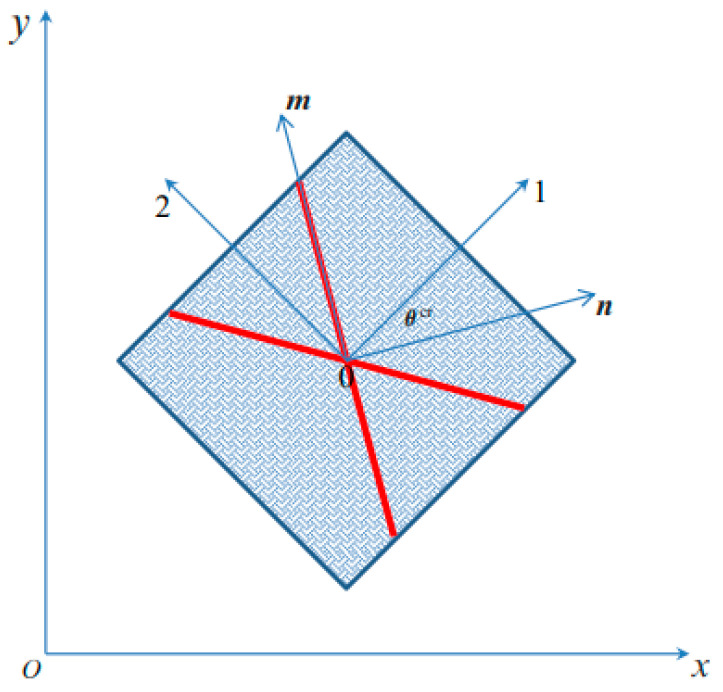
Definition of the localization angle $\theta_{\mathrm{cr}}$.

In this section, the orientation of the slip lines is analytically obtained for orthotropic and pressure-dependent plastic solids subjected to plane strain and plane stress conditions. The strain localization angle is measured counter-clockwise $\theta_{\mathrm{cr}}$ ∈ [$-\frac{\pi }{2},\frac{\pi }{2}$ ] as the angle between the vector n normal to the discontinuity and the material axis 1; see Figure 4.

Let (n,m,p) be the basis formed by the orthonormal vectors normal and tangential to the discontinuity S, such that vectors n and m are respectively normal and tangential to the trace of ***S*** in the reference plane *xy* and vector p points in the out-of-plane *z* direction, as shown in Figure 4.

The strain localization Equation (16) requires the flow tensor in Equation (31) to be written in this system. Let $\theta^{\mathrm{cr}}$ be the angle between the material system (1, 2, 3) and the (n,m,p) system. Then

(38) $$\begin{array}{c} \Lambda_{mm}=\Lambda_{11}\sin^{2}\theta^{\mathrm{cr}}+\Lambda_{22}\cos^{2}\theta^{\mathrm{cr}}+2\Lambda_{12}\sin\theta^{\mathrm{cr}}\cos\theta^{\mathrm{cr}}\\ \Lambda_{pp}=\Lambda_{33}\\ \Lambda_{mp}=0 \end{array}$$

The strain localization angle $\theta^{\mathrm{cr}}$ is obtained from the kinematic constraints in Equation (16), that is, equating these components to zero. Solving $\Lambda_{mm}\left(\theta^{\mathrm{cr}}\right)=0$ for $\tan\theta^{\mathrm{cr}}:$

(39) $$\tan\theta^{\mathrm{cr}}=-\frac{\Lambda_{12}}{\Lambda_{11}}\pm \sqrt{{\left(\frac{\Lambda_{12}}{\Lambda_{11}}\right)}^{2}-\frac{\Lambda_{22}}{\Lambda_{11}}}$$

As can be seen, the strain localization angle $\theta^{\mathrm{cr}}$ depends on the stress state upon strain localization. The condition $\Lambda_{pp}\left(\theta^{cr}\right)=\Lambda_{33}=0$ (38) needs to be imposed in plane stress and strain conditions.

**Remark** **7.** *For the case of $\Lambda_{12}=0$, where the no shear stress acts on the material axes, Equation (39) simplifies to*

(40) $$\tan\theta^{\mathrm{cr}}=\pm \sqrt{-\frac{\Lambda_{22}}{\Lambda_{11}}}$$

**Remark** **8.** *The kinematic constraints produce two alternative strain localization angles, see also Figure 3. The in-between angles that follow from Equation (40) are*

(41) $$\tan\left(\theta_{1}^{\mathrm{cr}}-\theta_{2}^{\mathrm{cr}}\right)=\frac{\tan\theta_{1}^{\mathrm{cr}}-\tan\theta_{2}^{\mathrm{cr}}}{1+\tan\theta_{1}^{\mathrm{cr}}\tan\theta_{2}^{\mathrm{cr}}}=\pm \left(\frac{2\sqrt{-\frac{\Lambda_{22}}{\Lambda_{11}}}}{1+\frac{\Lambda_{22}}{\Lambda_{11}}}\right)$$

**Remark** **9.** *In purely isochoric models (von Mises, Hill),* $\Lambda_{11}=-\Lambda_{22},$ *and* $\tan\left(\theta_{1}^{cr}-\theta_{2}^{cr}\right)=\pm \infty$ *, so* $\theta_{1}^{cr}-\theta_{2}^{cr}=\pm 90{}^{\circ}.$

**Remark** **10.** *The angle of the slip lines (counter-clockwise from 1-axis) is*$\theta^{slip}=\frac{\pi }{2}-\theta^{cr}$:

(42) $$\tan\theta^{\mathrm{slip}}={\left(\tan\theta^{\mathrm{cr}}\right)}^{-1}.\;$$

**Remark** **11.** *The above expressions are obtained for the stress expressed in the material system. These are obtained from the stresses in the global (x,y,z) system by standard transformation. For instance, in plane strain conditions*

(43) $$\left[\begin{array}{c} \sigma_{11}\\ \sigma_{22}\\ \sigma_{33}\\ \sigma_{12} \end{array}\right]=\left[\begin{array}{cccc} \cos^{2}\alpha & \sin^{2}\alpha & 0 & -2\cos\alpha \sin\alpha \\ \sin^{2}\alpha & \cos^{2}\alpha & 0 & 2\cos\alpha \sin\alpha \\ 0 & 0 & 1 & 0\\ \cos\alpha \sin\alpha & -\cos\alpha \sin\alpha & 0 & \cos^{2}\alpha -\sin^{2}\alpha \end{array}\right]\left[\begin{array}{c} \sigma_{xx}\\ \sigma_{yy}\\ \sigma_{zz}\\ \sigma_{xy} \end{array}\right]$$

where α is the tilt angle between the global axis *x* and the material local axis 1 measured counter-clockwise.

#### 3.3.1. Plane Stress

In plane stress, $\sigma_{33}=\sigma_{pp}=0$.

In this case, the non-zero plastic flow components $\Lambda_{ij}$ in Equation (40) are

(44) $$\begin{array}{c} \Lambda_{11}=\frac{q_{0}^{2}}{2\phi }\left[2\left(G+F\right)\sigma_{11}-2\tilde{F}\sigma_{22}+I\right]\\ \Lambda_{22}=\frac{q_{0}^{2}}{2\phi }\left[2\left(F+H\right)\sigma_{22}-2\tilde{F}\sigma_{11}+J\right]\\ \Lambda_{12}=\Lambda_{21}=\frac{q_{0}^{2}}{2\phi }L\sigma_{12} \end{array}$$

These components can be substituted in Equation (39).

#### 3.3.2. Plane Strain

In this case, the non-zero plastic flow components $\Lambda_{ij}$ in Equation (40) are considered with Equation (38).

(45) $$\begin{array}{c} \Lambda_{11}=\frac{q_{0}^{2}}{2\phi }\left[2\left(G+F\right)\sigma_{11}-2\tilde{F}\sigma_{22}-2\tilde{G}\sigma_{33}+I\right]\\ \Lambda_{22}=\frac{q_{0}^{2}}{2\phi }\left[2\left(F+H\right)\sigma_{22}-2\tilde{F}\sigma_{11}-2\tilde{H}\sigma_{33}+J\right]\\ \Lambda_{33}=\frac{q_{0}^{2}}{2\phi }\left[2\left(G+H\right)\sigma_{33}-2\tilde{G}\sigma_{11}-2\tilde{H}\sigma_{22}+K\right]\\ \Lambda_{12}=\Lambda_{21}=\frac{q_{0}^{2}}{2\phi }L\sigma_{12} \end{array}$$

From the kinematical condition $\Lambda_{\mathrm{pp}}\left(\theta^{\mathrm{cr}}\right)=\Lambda_{33}=0$, $\sigma_{33}$ is obtained as

(46) $$\sigma_{33}=\frac{2\left(\tilde{G}\sigma_{11}+\tilde{H}\sigma_{22}\right)-K}{2\left(G+H\right)}$$

and inserted into Equation (44), and these components can be then substituted in Equation (39).

### 3.4. Geometrical Interpretation of the Strain Localization Angle in the Stress Space

In the following, a geometrical interpretation of the strain localization angles obtained analytically is offered. As explained, Figure 3 gives a partial graphical representation of the orthotropic yield criteria projected into the HW space when the principal stresses act on the material axis, that is, no shear stress appears on the material system.

**Figure 5:**
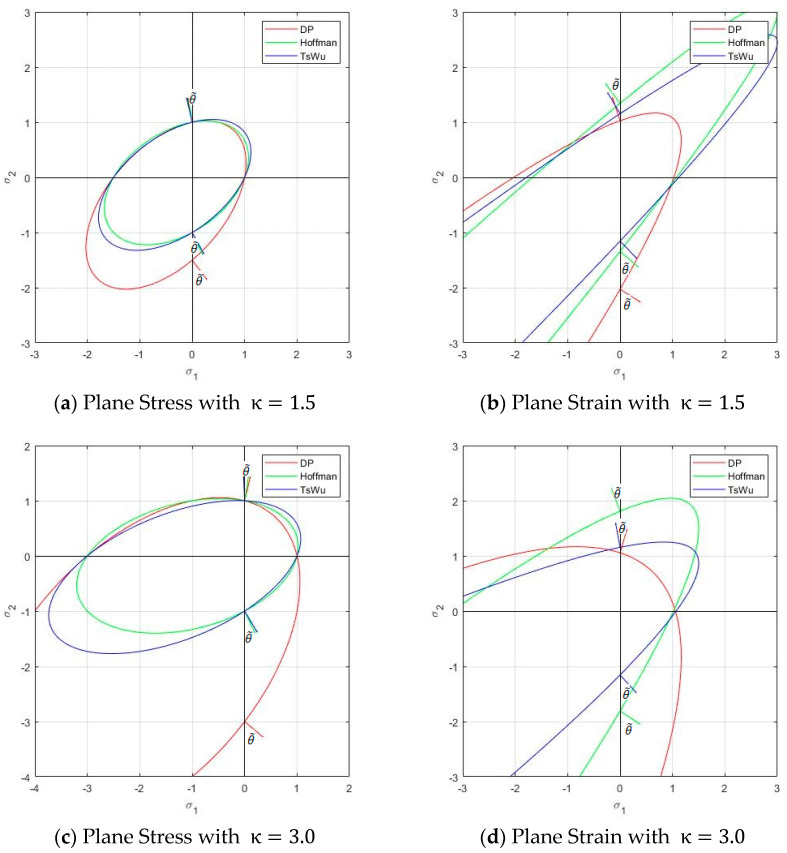
Cross sections of the yield criteria and strain localization angle under uniaxial tension and compression in plane stress and plane strain. (**a**) Plane stress with κ=1.5; (**b**) Plane strain with κ=1.5; (**c**) Plane stress with κ=3.0; (**d**) Plane Strain with κ=3.0.

In Figure 5, a cross section of those paraboloids by a horizontal plane is given. For plane stress, the plane $\sigma_{33}=0$ is used; for plane strain, the plane $\sigma_{33}=\left[2\left(\tilde{G}\sigma_{11}+\tilde{H}\sigma_{22}\right)-K\right]/\;2\left(G+H\right)$, from Equation (46), is used. The isotropic Drucker–Prager and the orthotropic Hoffmann and Tsai–Wu criteria are depicted for ratios $\kappa =f^{c}/\;f^{t}=1.5\;and\;3.0$ and $f_{2}^{c}\;/f_{2}^{t}=f_{3}^{c}\;/f_{3}^{t}=1;\;$all tensile strengths are taken equal to 1. For plane stress, the intersected quadratic curves are ellipses; for plane strain, they are parabolas. They are more stretched for higher ratios κ.

In Figure 5, the projection of the plastic flow vector, normal to the yield surface, for uniaxial tension and compression in the 2-direction, is also plotted. See the next section for the analytical values.

**Remark** **12.** *The angle*$\tilde{\theta }$ between this projected flow vector and the 2-axis is related to the strain localization angle $\theta^{cr},\;$because

(47) $$\tan\tilde{\theta }=-\frac{\Lambda_{22}}{\Lambda_{11}}=\tan^{2}\theta^{\mathrm{cr}}$$

### 3.5. Uniaxial Tension and Compression: Analytical Strain Localization Angles

In the following, the analytical values of the strain localization angle are obtained for the uniaxial tension and compression cases illustrated in Figure 5. Material strengths are those indicated in the previous section; with those, the coefficients for matrix P and vector Q are computed for the three different criteria (Drucker–Prager, Hoffmann and Tsai–Wu) and listed in Table 1.

#### 3.5.1. Plane Stress

For uniaxial tension in plane stress, the stress state is

(48) $$\sigma_{11}=0,\;\;\sigma_{22}=\sigma >0,\;\;\sigma_{12}=0,\;\;\sigma_{33}=0$$

Therefore, the kinematical condition $\Lambda_{33}=0$ (38) needs not be considered, and no extra constraint imposes on the stress state upon strain localization. Therefore, once the initial yield surface, Φ(σ,ζ)=0, is reached, strain localization occurs at the same instant, with the orientation determined from the corresponding flow tensor.

A stress $\sigma_{22}=\sigma =1$ is taken so that the point [0, σ*,* 0] is on the yield surface, see Figure 5a.

As $\Lambda_{12}=0,$

(49) $$\tan\theta^{\mathrm{slip}}={\left(\tan\theta^{\mathrm{cr}}\right)}^{-1}=\pm \sqrt{-\frac{\Lambda_{11}}{\Lambda_{22}}}=\pm \sqrt{\frac{2\tilde{F}\sigma_{22}-I}{2\left(F+H\right)\sigma_{22}+J}}$$

The obtained values for $\theta^{\mathrm{slip}}$ are given in Table 2. Results corresponding to uniaxial compression are also given in the Table. Note that the localization angles under tension and compression are very different for the various yield criteria, as depicted graphically in Figure 5.

**Remark** **13.** *Note that for $I=2\tilde{F}$ the localization angle $\theta^{cr}=0\;for\;tension,\;as\;\sigma_{22}=\sigma =1.$ This happens if the compressive strength is sufficiently larger than the tensile strength; for instance, it happens for the ratio κ=2 for the Drucker–Prager criterion. For larger ratios, there is no real value for the localization angles. In compression, for $\sigma_{22}=\sigma =-1$, this happens reciprocally, that is, if the compressive strength is sufficiently smaller than the tensile strength*.

#### 3.5.2. Plane Strain

In the plane strain case, the kinematical condition $\Lambda_{33}=0$ (38) needs to be enforced. From this,

(50) $$\sigma_{33}=\frac{2\left(\tilde{G}\sigma_{11}+\tilde{H}\sigma_{22}\right)-K}{2\left(G+H\right)}$$

A stress $\sigma_{22}=\sigma$ is found so that the point [0, σ, $\sigma_{33}$] is on the corresponding yield surface, see Figure 5a.

Then,

(51) $$\tan\theta^{\mathrm{slip}}={\left(\tan\theta^{\mathrm{cr}}\right)}^{-1}=\pm \sqrt{-\frac{\Lambda_{11}}{\Lambda_{22}}}=\pm \sqrt{\frac{2\tilde{F}\sigma_{22}+2\tilde{G\;}\sigma_{33}-I}{2\left(F+H\right)\sigma_{22}-2\tilde{H}\sigma_{33}+J}}.$$

The obtained values for $\theta^{\mathrm{slip}}$ are given in Table 3. Results corresponding to uniaxial compression are also given. Note that the angles under tension and compression are distinct, and they are also different for the various yield criteria, as shown in Figure 5.

## 4. Numerical Verification

In this section, FEM analyses are performed to numerically verify the analytical results obtained in Section 3 and derived from the strain localization analysis in Section 2.

It is emphasized that the numerical verification is totally independent of the analytical results. That is, the numerical analyses follow the standard procedure for solving the nonlinear mechanical problem and plastic behavior appears and evolves into the formation of slip lines; and the analytical results are not by any means used.

In previous works [45], it has been demonstrated that the strain localization angle is independent of the elastic properties. Therefore, the argument is not pursued here. Similarly, the localization angle does not depend on the softening behavior [47], so perfect plasticity is assumed in the following.

Although pressure-dependent plasticity models are to be investigated, they are compared to the isochoric von Mises model. To avoid volumetric locking in nearly incompressible situations, B-bar finite elements [49] are used in 2D and 3D.

### 4.1. B-Bar Finite Element

The B-bar element is a particular implementation of the mixed displacement/pressure *Q1P0* element in which the constant pressure has been eliminated at element level at the expense of renouncing the incompressible limit. This is accomplished by evaluating the constant mean stress in terms of the mean volumetric strain, the latter computed from the nodal displacements.

The standard discrete strain-displacement B matrix, computed at each integration point from the Cartesian derivatives of the nodal shape functions, is split into its volumetric and deviatoric parts

(52) $$B=B^{\mathrm{vol}}+B^{\mathrm{dev}}$$

A mean volumetric sub-matrix ${\bar{B}}^{\mathrm{vol}}$ is computed as

(53) $${\bar{B}}^{\mathrm{vol}}=\frac{1}{n_{g}}\overset{n_{g}}{\underset{k=1}{\sum }}B_{k}^{\mathrm{vol}}$$

where $n_{g}$ is the number of integration points in the element.

The B-bar discrete strain-displacement matrix is obtained as

(54) $$\bar{B}={\bar{B}}^{\mathrm{vol}}+B^{\mathrm{dev}}$$

The B-bar element has some zero-energy modes that may show as spurious hour-glassing in some instances. This may be avoided by using

(55) $${\bar{B}}^{stab}=\bar{B}+\left(1-\tau \right)\left[B^{\mathrm{vol}}-{\bar{B}}^{\mathrm{vol}}\right]$$

For τ=1, then ${\bar{B}}^{stab}={\bar{B}}^{\mathrm{vol}}+B^{\mathrm{dev}}=\bar{B}$ is identical to the B-bar formulation. For τ=0, then ${\bar{B}}^{stab}=B^{\mathrm{vol}}+B^{\mathrm{dev}}$ is identical to the standard formulation.

### 4.2. Uniaxial Tension and Compression: Numerical Verification

**Figure 6:**
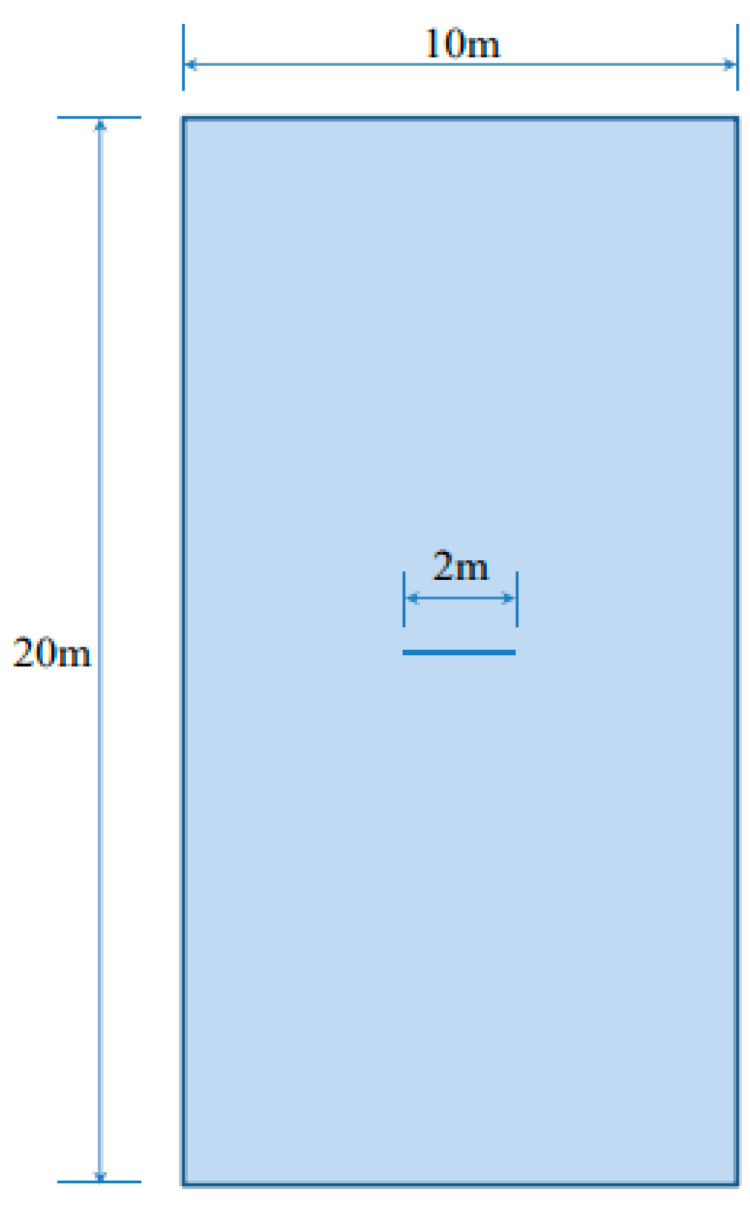
Geometry of a strip under vertical stretching.

In this section, the above B-bar finite element is used to perform benchmark verifications in strain localization analysis. The benchmark example is a strip loaded in uniaxial tension and compression via imposed vertical displacements at the top and bottom ends; the horizontal movement is not restrained. As shown in Figure 6, the strip has dimensions 10 m×20 m (width × height). A sharp horizontal slit (2 m) is inserted in the center of strip to introduce the perturbation necessary to trigger strain localization.

In this problem the stress field is known a priori. Plane strain and plane stress conditions are investigated. In both cases, the far field stress state corresponds exactly to those assumed for the analytical results in Section 3:

(56) $$\sigma_{11}=0,\;\;\sigma_{22}=\sigma >0,\;\;\sigma_{12}=0$$

The sharp horizontal slit causes a stress concentration that triggers the onset of plastic behavior and strain localization; subsequently, straight slip lines stem from these and cross the strip at well-defined slopes that must follow the angles analytically predicted in Section 2 and Section 3. The numerical results obtained in the FE analysis are used to validate the strain localization analysis in Section 2 and the analytical results in Section 3 that follow from it.

The following material properties are used: Young’s modulus $E=1.0\times 10^{7}\;\mathrm{MPa}$, Poisson’s ratio ν=0.2. Several orthotropic elasto-plastic criteria are compared; the different plastic yield strengths along the material axes are detailed for each case. Perfect plasticity is assumed.

Structured meshes of regular quadrilateral are employed. Square elements (0.05 m×0.05 m) are arranged 200 horizontally and 400 vertically, with a total of 80,000 elements used for plane strain 2D simulations. Plane stress cases are simulated in 3D, with as many hexahedral elements arranged in a mesh 1 element thick. In all cases, 500 time steps are performed to complete the analyses. The constitutive laws and finite elements used have been implemented in the COMET finite element program, developed by the authors at the International Center for Numerical Methods in Engineering (CIMNE). Pre- and post-processing are done with GiD, also developed at CIMNE.

#### 4.2.1. Isotropic Incompressible and Cohesive–Frictional Models

In this subsection, strain localization is first investigated for isotropic incompressible and pressure sensitive models.

Isotropic von Mises *J2* plasticity with yield strength $f=1.0\times 10^{4}\;\mathrm{MPa}$ is used as reference case. Insensitive to pressure, under plane strain, tensile and compression tests show the same localization angles (±45°), while under plane stress, the localization angles are ±35.26° from the horizontal axis, measured in a counter-clockwise manner.

Isotropic Parabolic Drucker–Prager models are also considered. A tensile strength $f^{t}=1.0\times 10^{4}\;\mathrm{MPa}$ and different compressive strengths according to the ratio κ= $f^{c}/\;f^{t}\;$are used, κ=1.25, 1.50 for tension, κ=2.0, 3.0 for compression; the isotropic shear strength is $\sqrt{f^{c}f^{t}\;/\;3}$.

**Figure 7:**
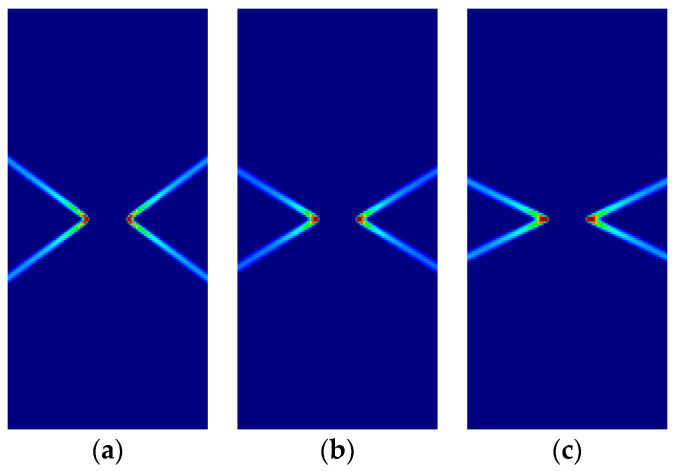
Strip under vertical plane stress tension (Parabolic Drucker–Prager): (**a**) κ=1.0, identical to von Mises; (**b**) κ=1.25; (**c**) κ=1.5.

**Figure 8:**
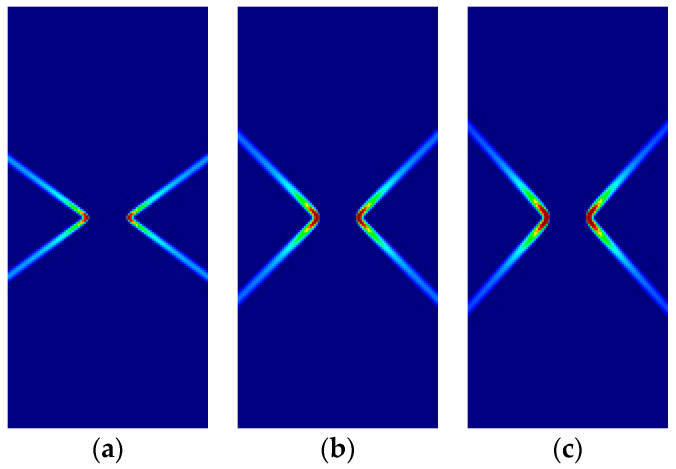
Strip under vertical plane stress compression (Parabolic Drucker–Prager): (**a**) κ=1.0, identical to von Mises; (**b**) κ=2.0; (**c**) κ=3.0.

**Figure 9:**
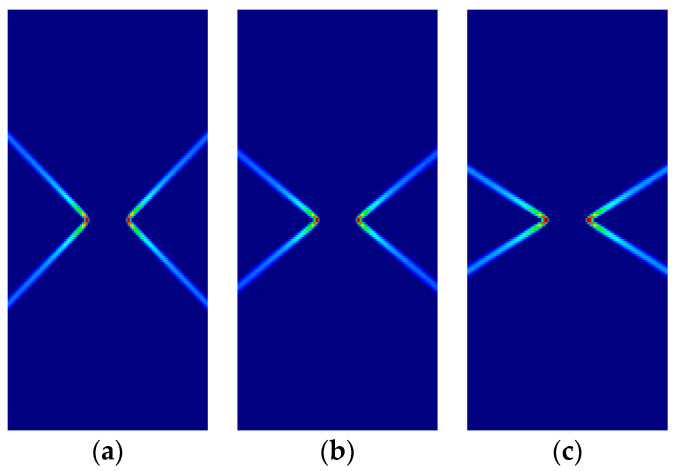
Strip under vertical plane strain tension (Parabolic Drucker–Prager): (**a**) κ=1.0, identical to von-Mises; (**b**) κ=1.25; (**c**) κ=1.5.

**Figure 10:**
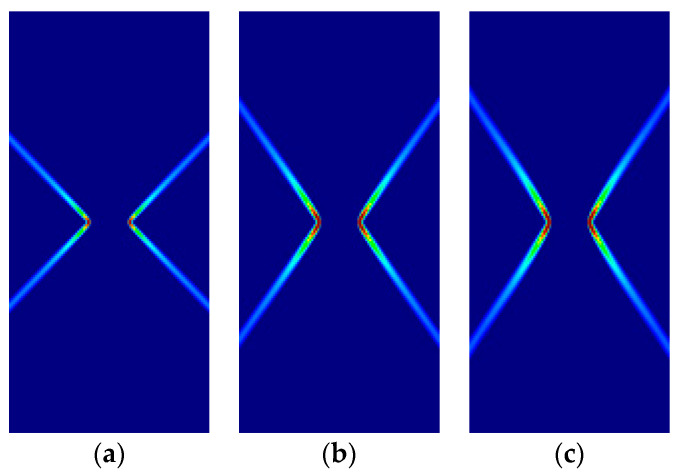
Strip under vertical plane strain compression (Parabolic Drucker–Prager): (**a**) κ=1.0 identical to von-Mises; (**b**) κ=2.0; (**c**) κ=3.0.

Some of the corresponding analytical results are given in Section 3.5. Here, the analytical and numerical results are presented for comparison in Figure 7, Figure 8, Figure 9 and Figure 10 and Table 4, Table 5, Table 6 and Table 7. Plane stress and plane strain results are shown both for tension and compression.

In the figures in this and the following sections, contour fills of the equivalent plastic strain are depicted to show the orientation of the slip lines and the corresponding failure mechanisms. The resolution of the mesh and the color pattern are selected so that these can be easily perceived. The red to blue color range indicates the largest to smallest magnitude of the equivalent plastic strain. For the numerical results, the numerical Lode angle is measured at the point in the slip line located 1 m to the right from the right end of the slit. The angle of the slide slip line is measured counter-clockwise from the *x*-axis.

For all cases, the numerical results are coincident with the analytical results. Correct angles of the slip lines are depicted in Figure 7, Figure 8, Figure 9 and Figure 10. Also, the coincidence between analytical and numerical results is shown in Table 4, Table 5, Table 6 and Table 7. The strain localization angle decreases with increasing ratios κ in the tensile tests (Figure 7 and Figure 9), while the strain localization angle increases with κ in the compressive tests (Figure 8 and Figure 10). The coincidence of the analytical and numerical Lode angles in the plane strain cases indicates that the kinematical constraint imposed by the $\Lambda_{33}=\Lambda_{zz}=0$ condition is verified.

#### 4.2.2. Isotropic and Orthotropic Cohesive–Frictional Models

In this subsection, the formation of slip lines is now investigated for orthotropic Hoffman and Tsai–Wu pressure-sensitive models and compared to the isotropic counterpart. The orthotropy material axes (1,2,3) are coincident with the global axes (*x,y,z*); relative tilting is investigated in Appendix B.

For the comparison, a ratio of compressive to tensile strengths κ=1.5 is taken for the tension tests and κ=3.0 for the compression tests. For the orthotropic models, all the yield strengths are taken as $f=1.0\times 10^{4}\;\mathrm{MPa}$, except the compressive $f_{x}^{c}$, which is taken to the κ ratio; shear strength $f_{xy}=\sqrt{f_{x}^{c}f_{y}^{c}\;/\;3}$.

**Figure 11:**
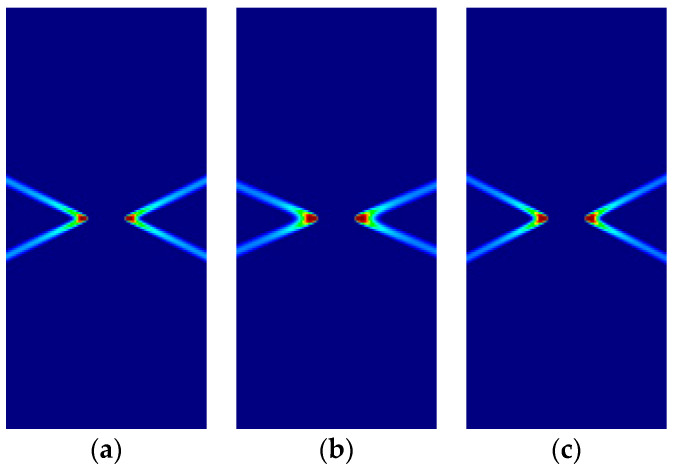
Strip under vertical plane stress tension (Cohesive–frictional models, κ=1.5): (**a**) Parabolic Drucker–Prager; (**b**) Hoffman; (**c**) Tsai–Wu.

**Figure 12:**
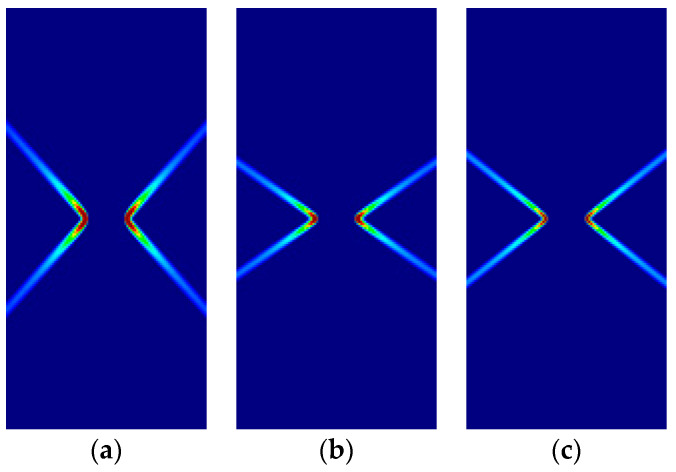
Strip under vertical plane stress compression (Cohesive–frictional models, κ=3.0): (**a**) Parabolic Drucker–Prager; (**b**) Hoffman; (**c**) Tsai–Wu.

**Figure 13:**
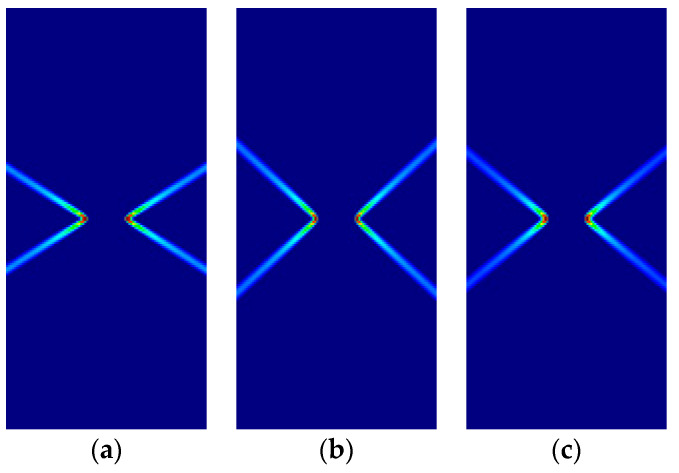
Strip under vertical plane strain tension (Cohesive–frictional models, κ=1.5): (**a**) Parabolic Drucker–Prager; (**b**) Hoffman; (**c**) Tsai–Wu.

**Figure 14:**
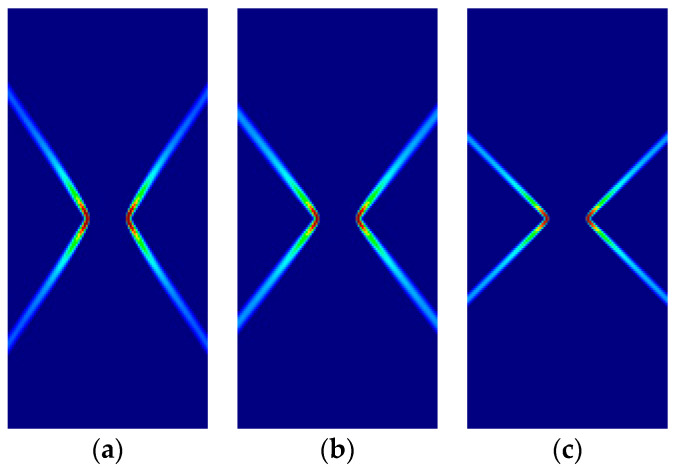
Strip under vertical plane strain tension (Cohesive–frictional models, κ=3.0): (**a**) Parabolic Drucker–Prager; (**b**) Hoffman; (**c**) Tsai–Wu.

Some of the corresponding analytical results are given in Section 3.5. In the following, the analytical and numerical results are presented for comparison in Figure 11, Figure 12, Figure 13 and Figure 14 and Table 8, Table 9, Table 10 and Table 11.

As previously, for all cases, the numerical and analytical results are coincident. Note that Hoffman and Tsai–Wu models produce different outcomes for the same material properties, as they use different$\;\tilde{F}$*,* $\tilde{G}$ and $\tilde{H}$ parameters. Lode angles in plane stress are 0° under tensile loading and 60° under compressive loading; they vary in plane strain.

### 4.3. Prandtl’s Punch Test

The second example is Prandtl’s punch test, a 2D plane problem in which a flat rigid die punches into an elasto-plastic semi-infinite medium. Classical solutions to this problem for rigid-plastic materials are well-known.

**Figure 15:**
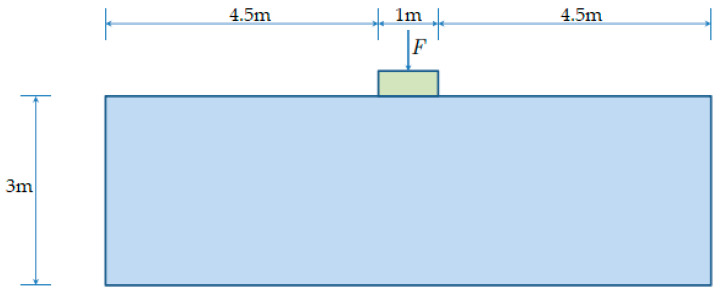
Geometry Prandtl’s punch test. The bottom edge is fixed in both directions, while the left and right edges are constrained along the horizontal direction.

As shown in Figure 15, the computational domain of the elasto-plastic medium is 10 m×3 m (width × height). Boundary conditions consist of a fixed bottom edge, left and right edges horizontally restrained. Punching is applied by imposing an increasing vertical displacement at the base of the rigid die; the horizontal movement is restrained at this base.

Material properties are the same as for the strip under vertical loading. A regular mesh of 192,000 (800 × 240) square B-bar elements (0.0125 m × 0.0125 m) is used. In all cases, 1000 time steps are performed to complete the analyses.

The mechanics of the failure are as follows. Plastic yielding starts at the singular points at the extreme ends of the rigid die. From here, two slip lines dig into the supporting elasto-plastic medium at diverging angles. Further loading causes the formation of a collapse mechanism in which the triangular wedge of material immediately under the punch moves vertically, causing the outward lateral displacement of adjoining material and the upwards displacement of the material located in the triangular wedges close to the surface and next to the flat punch.

**Figure 16:**
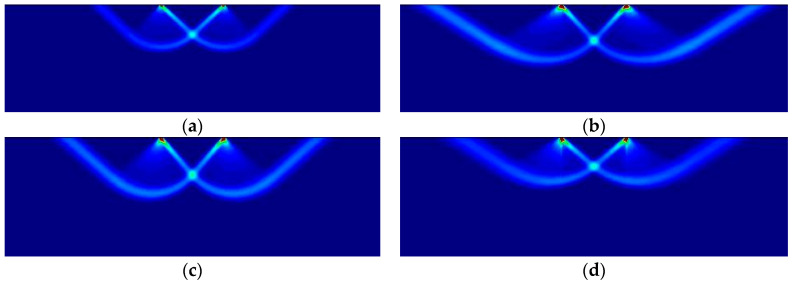
Prandtl’s punch test: (**a**) von Mises; (**b**) Parabolic Drucker–Prager, κ=3.0; (**c**) Hoffman, κ=3.0; (**d**) Tsai–Wu, κ=3.0.

Figure 16 shows the numerically obtained failure mechanisms for the four different cases studied, depending on the plastic criterion used in each one: (a) isotropic pressure-independent von Mises; (b) isotropic pressure-dependent Parabolic Drucker–Prager, κ=3.0; (c) orthotropic Hoffman, κ=3.0 in the horizontal direction; and (d) orthotropic Tsai–Wu, κ=3.0, in the horizontal direction. Associated perfect plasticity is used, so that the plastic potential coincides with the described yield criteria. The plots are zoomed in the region of interest, with identical magnification.

As can be observed, similar but notably different failure mechanisms form, depending on the plastic potential that applies. Although the process of the formation of the slip lines and the failure mechanism is analogous in all cases, the observed discrepancies in the slopes of the slip lines, and the corresponding amounts of mobilized material, depend on the plastic material properties.

**Figure 17:**
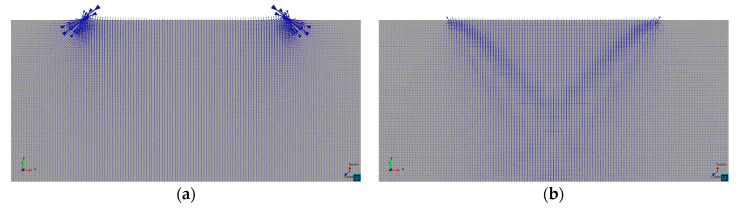
Directions of principal stresses below the rigid footing of Prandtl’s punch test (Parabolic Drucker–Prager, κ=3.0): (**a**) Elastic stage; (**b**) Final plastic stage.

Contrariwise to the case studied in the previous section, here the stress field is known a priori. Furthermore, substantial stress redistribution happens in the transition between the initial elastic stage and the final elasto-plastic state in which the failure mechanism is completely formed and yielding. This can be observed in Figure 17, where the distribution of the principal stresses in the elastic (initial) and plastic (stationary) states in the region below the punch are compared for the Drucker–Prager case (b). It can be seen that the stress state in the elastic range consists mainly of vertical stress $\sigma_{yy}$ and the corresponding out of plane $\sigma_{zz}$ (not shown in the figure), due to Poisson’s ratio ν=0.2. In contrast, in the stationary plastic stress state, in-plane horizontal $\sigma_{xx}$ have noticeably developed.

**Figure 18:**
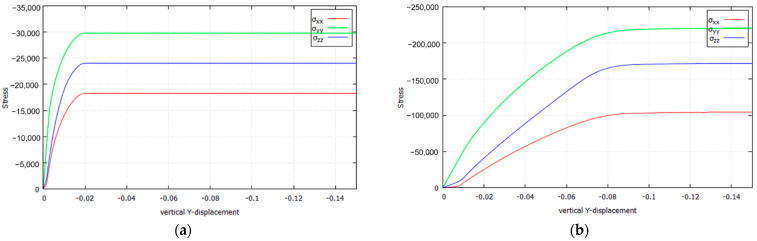

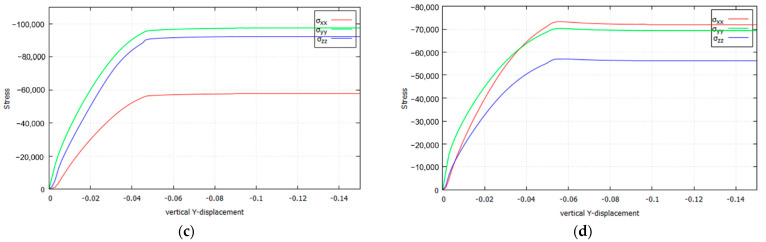
Stresses of the slide lines of Prandtl’s punch test (Plane Strain, κ=3.0): (**a**) von Mises; (**b**) Parabolic Drucker–Prager; (**c**) Hoffman; (**d**) Tsai–Wu.

**Figure 19:**
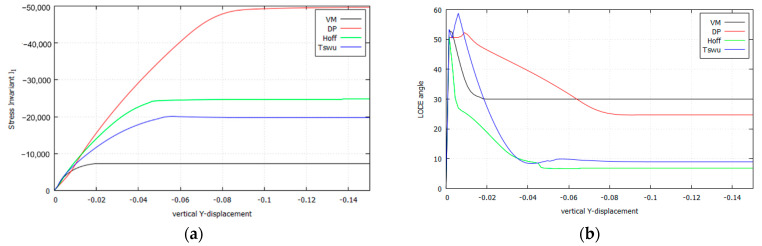
Results comparison where the slide lines cross (Plane Strain, κ=3.0: (**a**) Stress Invariant $I_{1}$; (**b**) Lode angle ϑ.

The extension and nature of this stress redistribution is further investigated in Figure 18, where the evolution of the normal stress components $\sigma_{xx},\;\sigma_{yy},\;\sigma_{zz}$ against the vertical displacement of the die is plotted for the four cases. The stresses are sampled at the corresponding crossing point of the slip lines, in the symmetry axis below the center of the punch. Figure 19 further summarizes the comparison of stress evolution by plotting the evolution of the stress Invariant $I_{1}$ and the Lode angle ϑ.

Several remarks are in order: (a) the extension of the transition phase largely differs from one case to the other; it is shorter for von Mises and longer for Drucker–Prager; (b) due to increasing vertical loading, the out of plane $\sigma_{zz}$ develops due to the plane strain constraint; (c) concurrently, the in-plane horizontal $\sigma_{xx}$ also develops, very much depending on the yield criterion; this later development shows the frictional and/or orthotropic nature of the plastic behavior.

It can be verified that this stress redistribution during the formation of the slip lines occurs precisely as dictated by the strain localization condition. This is done in Table 12 by comparing the value $\theta_{\mathrm{num}}^{\mathrm{slip}}$, measured directly from Figure 16, with the value $\theta_{\mathrm{ana}}^{\mathrm{slip}}$, obtained by applying the analytical condition (Section 3.3.2 and Section 3.5.2) to the numerically obtained values for the stresses. The correspondence between both value is remarkable.

## 5. Conclusions

In this work, the strain localization analysis of cohesive–frictional elasto-plastic materials is addressed, which applies to both strong and regularized slip lines and surfaces. Maxwell kinematics, stress boundedness and plastic consistency are invoked to derive the necessary strain localization conditions. Contrariwise to the usually studied conditions for strain bifurcation, these proffer requirements that do not depend on the elastic properties of the medium, but only on the plastic flow provided by the adopted plastic potential.

Expanding on previous works, application of the above localization conditions to isotropic and orthotropic cohesive–frictional plastic models derives analytical solutions for the strain localization angle and the slopes of the ensuing slip lines. The distinct effects of compressive and tensile loading are also evaluated.

The analytical results are validated independently by 2D plane stress and plane strain FE simulations using the B-bar element; namely, a strip under vertical tension and compression tests and Prandtl’s punch problem are investigated. In the first problem, the far field stress state is known, and the analytical results can be verified directly from the numerical simulations. In the second problem, once the failure mechanism and the corresponding stress field are computationally evaluated, these are shown to conform precisely with those anticipated by the strain localization condition.

## Figures and Tables

**Table 1:** Coefficients for matrix P and vector Q (κ=1.5)**.**

κ=1.5	F	G	H	$\tilde{F}$	$\tilde{G}$	$\tilde{H}$	I	J	K
DP	1/3	1/3	1/3	*F*	*G*	*H*	1/3	1/3	1/3
Hoffman	1/3	1/3	2/3	*F*	*G*	*H*	1/3	0	0
Tsai–Wu	1/3	1/3	2/3	$\sqrt{1/6}$	$\sqrt{1/6}$	0.5	1/3	0	0

**Table 2:** Plane Stress: stress state and slip-line angles for uniaxial tension and compression.

κ=1.5	Tension			Compression		
	$\sigma_{22}$	$\sigma_{33}$	$\theta^{slip}$	$\sigma_{22}$	$\sigma_{33}$	$\theta^{slip}$
DP	1.0000	0.0000	±24.0948°	−1.0000	0.0000	±41.8103°
Hoffman	1.0000	0.0000	±22.2077°	1.0000	0.0000	±35.2644°
Tsai–Wu	1.0000	0.0000	±26.1746°	−1.0000	0.0000	±37.1705°

**Table 3:** Plane Strain: stress state and slip-line angles for uniaxial tension and compression.

κ=1.5	Tension			Compression		
	$\sigma_{22}$	$\sigma_{33}$	$\theta^{slip}$	$\sigma_{22}$	$\sigma_{33}$	$\theta^{slip}$
DP	1.0275	0.2638	±30.4411°	−2.0275	−1.2638	±52.1384°
Hoffman	1.3416	0.8944	±41.3843°	−1.3416	−0.8944	±47.8857°
Tsai–Wu	1.1547	0.5774	±38.3075°	−1.1547	−0.5774	±45.1276°

**Table 4:** Analytical and numerical Lode and strain localization angles for isotropic models under plane stress tension.

κ	$\vartheta_{ana}$	$\vartheta_{num}$	$\theta_{ana}^{slip}$	$\theta_{num}^{slip}$
VM 1.0	0.0000°	0.6459°	35.2644°	35.4699°
DP 1.25	0.0000°	0.3803°	30.0000°	30.4342°
DP 1.5	0.0000°	0.7800°	24.0948°	24.2277°

**Table 5:** Analytical and numerical Lode and strain localization angles for isotropic models under plane stress compression.

κ	$\vartheta_{ana}$	$\vartheta_{num}$	$\theta_{ana}^{slip}$	$\theta_{num}^{slip}$
VM 1.0	60.0000°	59.3541°	35.2644°	35.4699°
DP 2.0	60.0000°	59.6989°	45.0000°	45.0000°
DP 3.0	60.0000°	59.6006°	48.1897°	48.8141°

**Table 6:** Analytical and numerical Lode and strain localization angles for isotropic models under plane strain tension.

κ	$\vartheta_{ana}$	$\vartheta_{num}$	$\theta_{ana}^{slip}$	$\theta_{num}^{slip}$
VM 1.0	30.0000°	30.1669°	45.0000°	45.0000°
DP 1.25	22.3378°	23.6244°	38.2626°	39.0939°
DP 1.5	14.3077°	15.9519°	30.4411°	31.4875°

**Table 7:** Analytical and numerical Lode and strain localization angles for isotropic models under plane strain compression.

κ	$\vartheta_{ana}$	$\vartheta_{num}$	$\theta_{ana}^{slip}$	$\theta_{num}^{slip}$
VM 1.0	30.0000°	30.1669°	45.0000°	45.0000°
DP 2.0	19.1066°	19.6359°	54.7356°	54.2934°
DP 3.0	17.1330°	17.6788°	56.6531°	57.5289°

**Table 8:** Analytical and numerical Lode and strain localization angles for frictional–cohesive models under plane stress tension, κ=1.5.

κ=1.5	$\vartheta_{ana}$	$\vartheta_{num}$	$\theta_{ana}^{slip}$	$\theta_{num}^{slip}$
Drucker–Prager	0.0000°	0.7800°	24.0948°	24.2277°
Hoffman	0.0000°	0.5258°	22.2077°	22.1355°
Tsai–Wu	0.0000°	0.3457°	26.1746°	26.5651°

**Table 9:** Analytical and numerical Lode and strain localization angles for frictional–cohesive models under plane stress compression, κ=3.0.

κ=3.0	$\vartheta_{ana}$	$\vartheta_{num}$	$\theta_{ana}^{slip}$	$\theta_{num}^{slip}$
Drucker–Prager	60.0000°	59.6006°	48.1897°	48.8141°
Hoffman	60.0000°	58.6061°	35.2644°	35.4699°
Tsai–Wu	60.0000°	59.8503°	38.2620°	37.7757°

**Table 10:** Analytical and numerical Lode and strain localization angles for frictional–cohesive models under plane strain tension, κ=1.5.

κ=1.5	$\vartheta_{ana}$	$\vartheta_{num}$	$\theta_{ana}^{slip}$	$\theta_{num}^{slip}$
Drucker–Prager	14.3077°	15.9519°	30.4411°	31.4875°
Hoffman	40.8934°	42.5043	41.3843°	41.5891°
Tsai–Wu	30.0000 °	30.7240°	38.3075°	38.2204°

**Table 11:** Analytical and numerical Lode and strain localization angles for frictional–cohesive models under plane strain compression, κ=3.0.

κ=3.0	$\vartheta_{ana}$	$\vartheta_{num}$	$\theta_{ana}^{slip}$	$\theta_{num}^{slip}$
Drucker–Prager	17.1330°	17.6788°	56.6531°	57.5288°
Hoffman	8.9483°	7.8626°	51.6975°	50.7106°
Tsai–Wu	30.0000°	29.4419°	44.4488°	44.6397°

**Table 12:** Stresses and localization angle in Prandtl’s punch test.

κ=3.0	$\sigma_{xx}$	$\sigma_{yy}$	$\sigma_{zz}$	$\vartheta_{num}\;$	$\theta_{num}^{slip}\;$	$\theta_{ana}^{slip}\;$
VM	−18286	−29832	−24061	29.9902°	45.0000°	45.0000°
Drucker–Prager	−104260	−220560	−171920	24.6035°	50.1944°	49.9512°
Hoffman	−57783	−97541	−92394	6.8372°	48.9909°	48.4646°
Tsai–Wu	−72007	−69365	−56219	8.9246°	41.1859°	40.6354°

**Figure A1:**
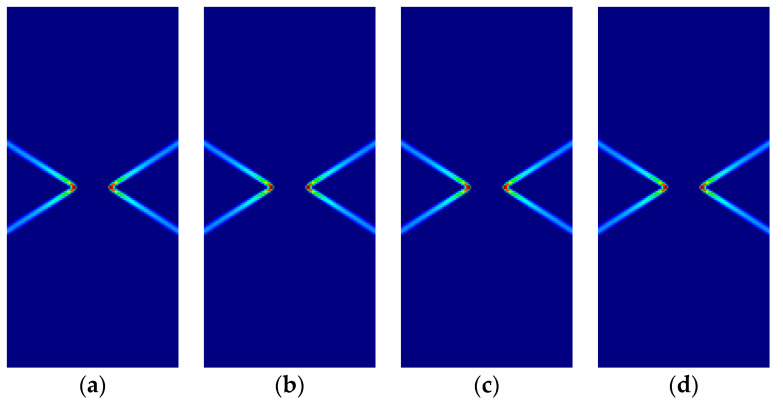
Strip under vertical plane strain tension (Parabolic Drucker–Prager, κ=1.5): (**a**) α=0°; (**b**) α=30°; (**c**) α=45°; (**d**) α=60°.

**Figure A2:**
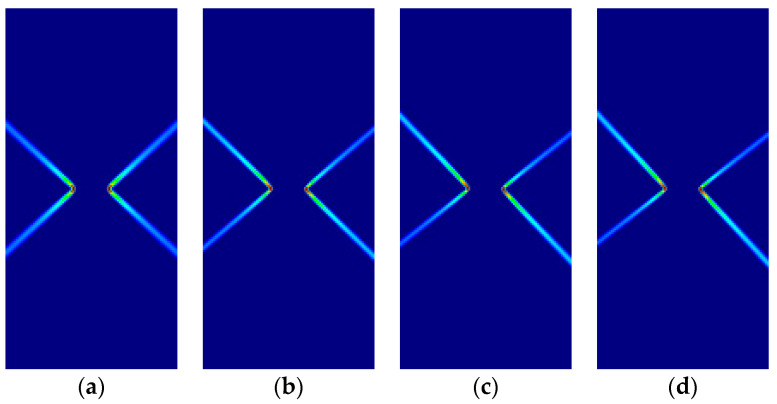
Strip under vertical plane strain tension (Hoffman, κ=1.5): (**a**) α=0°; (**b**) α=30°; (**c**) α=45°; (**d**) α=60°.

**Figure A3:**
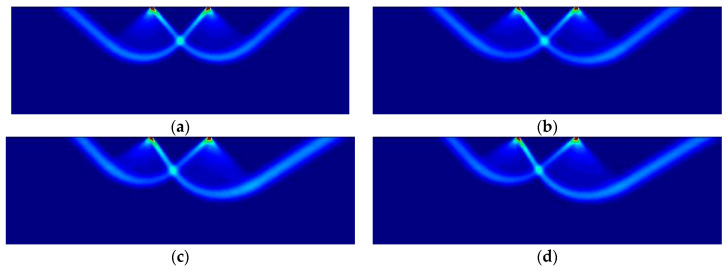
Prandtl’s punch test (Plane Strain, Hoffman, κ=3.0): (**a**) α=0°; (**b**) α=30°; (**c**) α=45°; (**d**) α=60°.

## Data Availability

The data presented in this study are available on request from the corresponding author.

## Appendix A. Stress Invariants and Lode Angle

The first, second, and third invariants of the stress tensors are

(A1) $$I_{1}=\sigma_{11}+\sigma_{22}+\sigma_{33},$$

(A2) $$I_{2}=\sigma_{11}\sigma_{22}+\sigma_{22}\sigma_{33}+\sigma_{11}\sigma_{33}-\sigma_{12}^{2}-\sigma_{23}^{2}-\sigma_{31}^{2},$$

(A3) $$I_{3}=\sigma_{11}\sigma_{22}\sigma_{33}+2\sigma_{12}\sigma_{23}\sigma_{31}-\sigma_{12}^{2}\sigma_{33}-\sigma_{23}^{2}\sigma_{11}-\sigma_{31}^{2}\sigma_{22}.$$

The second and third invariants of the deviatoric stress tensors are

(A4) $$J_{2}=\frac{1}{3}I_{1}^{2}-I_{2},$$

(A5) $$J_{3}=\frac{2}{27}I_{1}^{3}-\frac{1}{3}I_{1}I_{2}+I_{3}.$$

Lode Angle (positive cosine) $\left[0\leq \vartheta \leq \frac{\pi }{3}\right]$ is defined from these deviatoric stress invariants as

(A6) $$\vartheta =\frac{1}{3}\arccos\left(\frac{J_{3}}{2}{\left(\frac{3}{J_{2}}\right)}^{3/2}\right).$$

## Appendix B. Tilting of the Material Axes with Respect the Global Axes

In this Appendix, the effect of the tilting of the orthotropy material axes with respect the global axes and the orientation of the loading is demonstrated. The tilt angle α is measured counter-clockwise between the global *x* and the material 1 axes. The rotation transformation matrix was introduced in Remark 3.6.

Figure A1 and Table A1 show the results for the strip under vertical plane strain tension (Parabolic Drucker–Prager, κ = 1.5) and different tilting; as the model is isotropic, the results are insensitive to the rotation of the material axes.

**Table A1:** Analytical and numerical Lode and strain localization angles for Parabolic Drucker–Prager under plane strain tension, κ=1.5.

α	$\vartheta_{ana}$	$\vartheta_{num}$	$\theta_{ana}^{slip}$	$\theta_{num}^{slip}$
0°	14.3077°	15.9519°	30.4411°	31.4875°
30°	14.3077°	15.9519°	30.4411°	31.4875°
45°	14.3077°	15.9519°	30.4411°	31.4875°
60°	14.3077°	15.9519°	30.4411°	31.4875°

Figure A2 and Table A2 show corresponding results for the orthotropic Hoffman model, κ=1.5. Here, the effect of the tilting of the material axes is evident. Analytical and numerical results coincide for all slip lines.

**Table A2:** Analytical and numerical Lode and strain localization angles for Hoffman under plane strain tension, κ=1.5.

α	$\vartheta_{ana}$	$\vartheta_{num}$	$\theta_{ana}^{slip}$	$\theta_{num}^{slip}$
0°	40.8934°	42.5043°	41.3843°, 41.3843°	41.5891°, 41.5891°
30°	35.4964°	35.5087°	39.4100°, 43.0613°	39.5226°, 43.1524°
45°	30.0000°	30.0031°	37.4491°, 45.0000°	37.7757°, 45.0000°
60°	24.5036°	24.8814°	36.9016°, 45.7565°	36.8699°, 45.7073°

Finally, Figure A3 shows the effect of the tilting of the material axes in Prandtl’s punch test (Plane Strain, Hoffman model, κ=3.0).
